# What's in a Name? Not All Mesopredators Are Mesocarnivores

**DOI:** 10.1002/ece3.72768

**Published:** 2025-12-29

**Authors:** Emily K. Madsen, Lucy Eckersley, Jennifer F. Linden, Sandra Lai, Darragh Hare, David W. Macdonald, David Kimaili, Salum Kulunge, Yolanda Mutinhuma, Lisanne Petracca, Betty J. Rono, Lovemore Sibanda, Claudio Sillero‐Zubiri, Jessica Tacey, Femke Broekhuis

**Affiliations:** ^1^ Wildlife Conservation Research Unit, Department of Biology University of Oxford Oxford UK; ^2^ Royal Society of Biology London UK; ^3^ Institute of Zoology, Zoological Society of London London UK; ^4^ Department of Natural Resources and the Environment Cornell University Ithaca USA; ^5^ Department of Sociology and Anthropology South Eastern Kenya University Kitui Town Kenya; ^6^ Department of Wildlife Management Sokoine University of Agriculture Morogoro Tanzania; ^7^ Tanzania Wildlife Management Authority Morogoro Tanzania; ^8^ Centre for Sustainability Transitions Stellenbosch University Stellenbosch South Africa; ^9^ African Wildlife Economy Institute Stellenbosch University Stellenbosch South Africa; ^10^ Caesar Kleberg Wildlife Research Institute Texas A&M University Kingsville USA; ^11^ Department of Zoology and Entomology Rhodes University Grahamstown South Africa; ^12^ Department of Natural Resources Egerton University Njoro Kenya; ^13^ Cheetah Conservation Project Dete Zimbabwe; ^14^ Wildlife Ecology and Conservation Group Wageningen University and Research Wageningen the Netherlands

## Abstract

Ecological terms like *mesopredator* and *mesocarnivore* have distinct meanings, the former denoting trophic rank, the latter diet composition. Yet these terms are frequently conflated, leading to conceptual ambiguity. We argue for returning to original definitions and advocate for context‐sensitive, precise language to improve clarity and accuracy in scientific communication about fundamental ecological characteristics of species.

## Graphical abstract

Ecological terms like *mesopredator* and *mesocarnivore* have distinct meanings, the former denoting trophic rank, the latter diet composition, yet are frequently conflated, leading to conceptual ambiguity. We argue for returning to original definitions and advocate for context‐sensitive, precise language to improve clarity and accuracy in scientific communication about fundamental ecological characteristics of species.

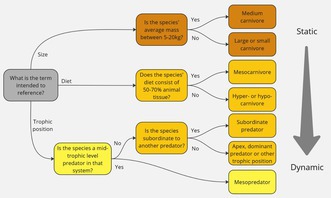

## 1 Introduction

Not all mesopredators are mesocarnivores, and not all competitively subordinate predators are mesopredators. Terms like *mesopredator* or *mesocarnivore* are not only academically meaningful labels; they can affect public understanding of species' ecological roles and may even influence research priorities and funding (Brodie et al. 2018; Eitzel et al. 2017). Using terms that group species based on shared traits enables concise and precise communication about those traits, and terminology can shape how species are prioritised in conservation and management policies.

For example, apex predators are often thought to exert broad, ecosystem‐wide effects, a topic that has been extensively studied (Kuijper et al. 2016; Haswell et al. 2017; Hollings et al. 2014; Schmitz et al. 2000), whereas there is less research on mesopredators having similarly far‐reaching impacts (Marneweck et al. 2022; Prugh et al. 2009). Consequently, a species labelled as an apex predator may receive higher conservation priority than an equally threatened species classified as a mesopredator (Ordiz et al. 2013). Precise understanding and usage of these types of terms is therefore important to ensure that the fundamental ecological characteristics of the species in question are accurately represented.

Yet, the terms mesopredator and mesocarnivore are regularly, but incorrectly, used interchangeably, risking misunderstanding, misinterpretation of fundamental theory, inaccurate evaluation of data outcomes, misplaced generation of new hypotheses, distortion of policy priorities and, at the extreme, public disagreement that could detract from a clear understanding of research results and thereby undermine their implementation. For example, the disagreement between Wolf et al. (2018) and (Miranda 2018) over the term ‘large carnivore’ used in Wolf and Ripple (2018) illustrates how inconsistent terminology can undermine the interpretation of research findings by creating confusion over key ecological concepts and the validity of the study's conclusions, as Miranda argued that the term was applied too broadly to include species that do not fit established ecological definitions of large carnivores. Precise, standardised language ensures effective communication within and beyond the scientific community. This is especially important as science becomes increasingly interdisciplinary and multilingual. Inconsistent terminology is, by definition, the enemy of clarity and risks alienating audiences by requiring repeated clarifications (Barrett et al. 2022).

There is considerable ambiguity in how the term ‘carnivore’ is used: as a common noun, it refers to any species which consumes animal tissue, regardless of taxonomy. However, taxonomically, the term when capitalised as a proper noun (Carnivores) relates specifically to members of the mammalian order Carnivora, also referred to as carnivorans. Here, we draw on our experience with Carnivores to illustrate our argument, though the principles we discuss apply to predators across taxonomic groups, as they occupy multiple trophic levels. Ecologists often categorise species by trophic position, dietary composition and body size (e.g., Carbone et al. 2007; Do Linh San et al. 2022). Size‐based groupings are static and relatively straightforward within Carnivora; large Carnivores are usually considered to be those weighing more than 20 kg (Carbone et al. 2007), although there is still ambiguity about thresholds for other more‐or‐less arbitrary size groupings and their usefulness given that within‐group diversity can be questioned. While the use of size‐based predator classification is well established in mammals, and to some extent in birds (e.g., Lyly et al. 2015), such frameworks are rarely applied to reptilian predators. This is potentially due to there being no clear thresholds between reptiles based on their size, though this could also be a reflection of a historical underrepresentation of reptiles in trophic ecology research, rather than a lack of ecological relevance (de Miranda 2017). Size‐based groupings also become less useful when discussing cross‐taxon interactions such as predation (e.g., Hatfield et al. 2024).

Although the term apex predator can be somewhat ambiguous due to context‐dependent trophic dynamics, we use it here for clarity to describe species occupying the highest trophic positions in their system which can shift species depending upon local assemblage. As a result of this higher trophic position, these apex species are typically competitively dominant over other predators at lower trophic levels. In this context, competitive dominance refers to hierarchies in resource competition, in which one species consistently gains greater access to limiting resources than its competitor; subsequently, a lower‐tiered competitively subordinate species. Carnivore species in the second highest tier of a food chain are regularly referred to as mesocarnivores and mesopredators. The term mesocarnivore first appeared on the Web of Science (all search fields) in 2000 referencing diet, 12 years after the term mesopredator in 1988. Both terms increased in usage since 2010, although both may have appeared earlier in articles which do not have their main text available online. These terms are often used interchangeably to describe Carnivores, and other predators, despite having ecological meanings that were originally distinct (e.g., Curveira‐Santos et al. 2021; Avrin et al. 2023; Roemer et al. 2009; Gigliotti et al. 2020). This incorrect usage is most likely the result of people not being aware of the original distinctions and can lead to conceptual and contextual ambiguity, potentially resulting in misleading or confusing interpretations.

## 2 What Is a Mesopredator Compared to a Mesocarnivore?

The term mesopredator lacks a strict biological definition but was first attributed to Larry Harris in 1987 in reference to ‘mesopredator release’ (Soulé et al. 1988). That usage, although not then precisely defined, was in the context of grey fox ( *Urocyon cinereoargenteus* ) populations increasing in California, USA, seemingly in response to a decline of coyotes ( *Canis latrans* ), the local apex predator in that area at the time. This original usage frames mesopredators as middle‐tier predators whose populations can expand, and sometimes adapt to occupy a higher niche (Prugh et al. 2009) when limiting apex predators (i.e., dominant competitors) are removed. But how do we define middle‐tier predators? Even among our authors, we discovered differences in default interpretations.

Soulé et al. (1988) referred to a mesopredator as a middle species positioned between an apex predator and the mesopredator's prey. However, this is inconsistent from its purely linguistic meaning, which would assume that a middle (from meso—the Greek word for middle) was situated between two ends, indicating a three‐tier predator system: mesopredators positioned between apex predators above and a third‐tier predator below. Whether Soulé et al. (1988) intended a three‐tiered usage (apex, middle and lower predators), or had different three tiers in mind in which mesopredators were any of those situated between apex predators and prey, is unclear. These distinctions might be trivial, had not the terms become so widely, and inconsistently, used, leading now to ambiguity with potentially important consequences. Levi and Wilmers (2012) for instance, discussed mesopredator release in the context of a four‐species chain (apex predator–mesopredator–lower predator–prey), which would align with this more linguistic interpretation. Whilst both interpretations intend to reference the trophic position of the Carnivore in question, the linguistic interpretation (indicating a three‐tier predator system) is less prone to confusion.

Alternatively, some interpret the term mesopredator more flexibly, using it to describe any predators that are competitively subordinate to another predator in the context of a specific question. In this case, if a study focused on only two Carnivores, but in that system they were both competitively subordinate to a third, for exmaple, red foxes ( *Vulpes vulpes* ) and coyotes relative to grey wolves ( *Canis lupus* ), the red fox might be referred to as the mesopredator on the grounds that it is subordinate to the coyote, the pair being studied, even if both are subordinate (and in some sense therefore meso‐) relative to the wolf, which is not part of the study. All three usages are context‐dependent, but the context, in the case of the first two (Soulé et al. 1988; Levi and Wilmers 2012), is the whole ecosystem, whilst the latter is framed by the specific relationships being studied.

Due to its deceptive similarity, some people also use the term mesocarnivore to refer to mid‐sized and mid‐tiered predators. However, mesocarnivore is defined in the Biology Online dictionary (BiologyOnline 2025) as ‘a carnivorous animal in which 50%–70% of its diet is flesh or meat of another animal’ and therefore is a form of omnivory. Van Valkenburgh (1989) is often credited with this definition (e.g., Meloro et al. (2015)), although they merely applied these group thresholds without using the term mesocarnivore. Mesocarnivores are contrasted with hypercarnivores (> 70% meat diet) and hypocarnivores (< 50% meat), both of which remain in customary usage in diet research. Mesocarnivore is therefore generally a static categorisation based on what a species eats; whilst there can be intraspecific and seasonal variation, there is a limit as diet is a fundamental attribute of a species, reflecting the limits to its adaptation and expressed in its fundamental niche (Van Valkenburgh 2007).

**FIGURE 1:**
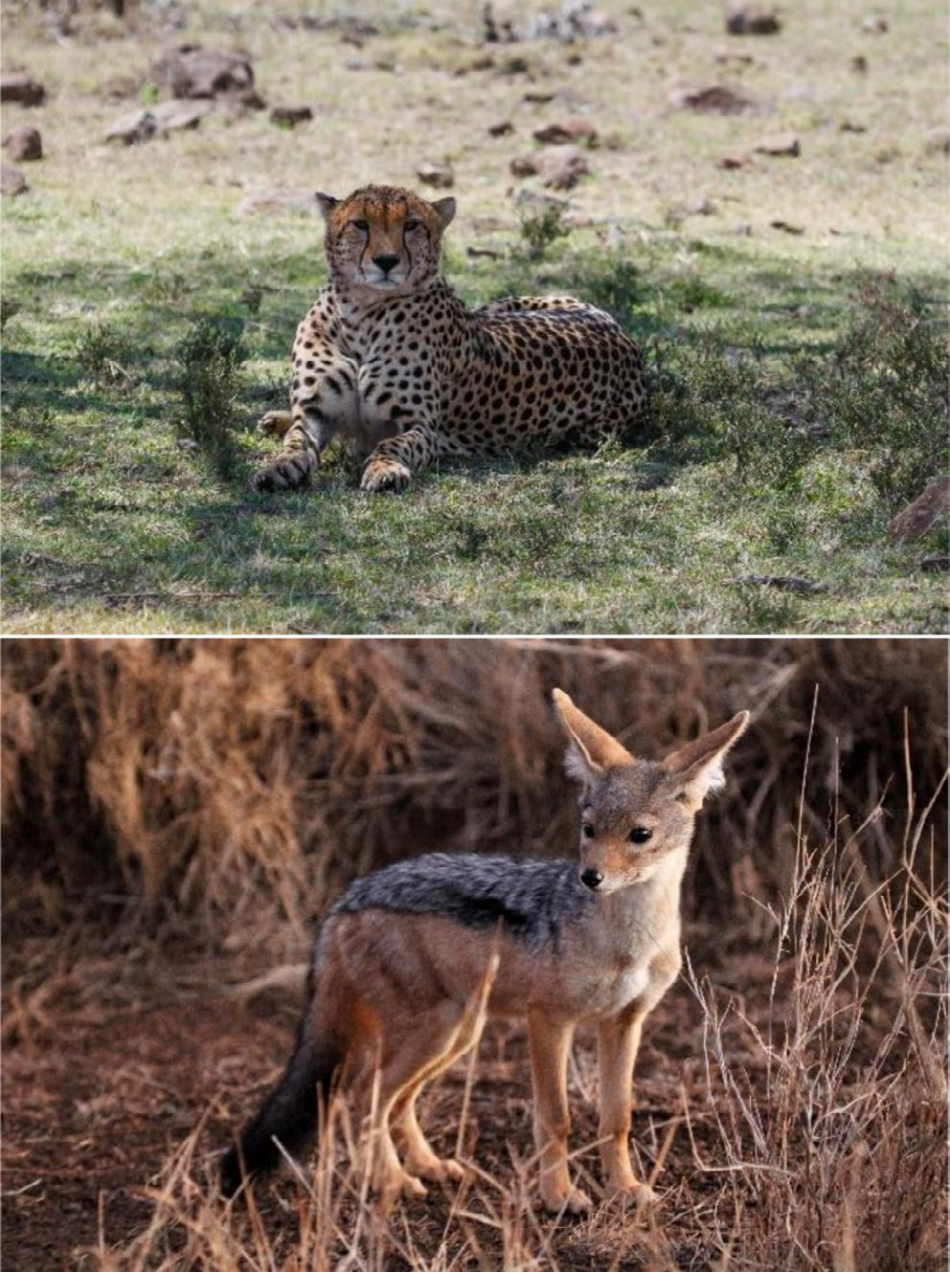
Top cheetah ( *Acinonyx jubatus* ) and bottom black‐backed jackal (Lupulella mesomelas). Whilst both have been called mesopredator and mesocarnivore in published papers, going by the original definitions of both terms, they are only applicable to black‐backed jackal. (Photograph credits: Emily Madsen).

Given the definition of mesocarnivore (an intermediate proportion of animal tissue in its diet), and the linguistic meaning of mesopredator (a mid‐tiered predator situated between an apex predator and third‐tiered predator), a mesocarnivore of any size or taxa (e.g., American badger *Taxidea taxus* ) would be unlikely to fill the ecological niche of a hypercarnivorous apex predator (e.g., mountain lion *Puma concolor* ), though a mesocarnivore may become the apex species in that system. In contrast, a hypercarnivorous mesopredator (e.g., bobcat *Lynx rufus* ) might be better suited to fill the niche of the hypercarnivorous apex predator. Additionally, if the term mesopredator is reflective of a species' position within an entire guild, then a large Carnivore should not be considered a mesopredator just because it is subordinate to another large Carnivore. On these grounds, a cheetah ( *Acinonyx jubatus* ; Figure 1) could not be described as a mesocarnivore, as all felids are obligate carnivores (i.e., > 70% of its diet is animal tissue; Yoshimura et al. 2021) and therefore hypercarnivores. Nor should it be considered a mesopredator—as if you were to divide any of its contemporary ecosystems, which include over 20 sympatric Carnivores, into three predator trophic levels, the cheetah would be placed into the top trophic level based upon its ecological niche and ecological impacts. However, within the same ecosystem, a black‐backed jackal (*Lupulella mesomelas*) could be both a mesocarnivore and a mesopredator (Klare et al. 2011).

Despite its conceptual appeal, applying the linguistic mesopredator framework to complex systems with high predator diversity such as African savannahs may be arbitrary, and even more so for marine and invertebrate predator systems. Categorising species into linear trophic tiers risks oversimplifying the intricate and context‐dependent nature of ecological interactions and food web dynamics. Accordingly, terms developed in one ecosystem may not translate well to others, and opting not to use them, or using alternative terminology, may offer greater ecological accuracy.

## 3 How Should These Terms Be Used Going Forwards?

**FIGURE 2:**
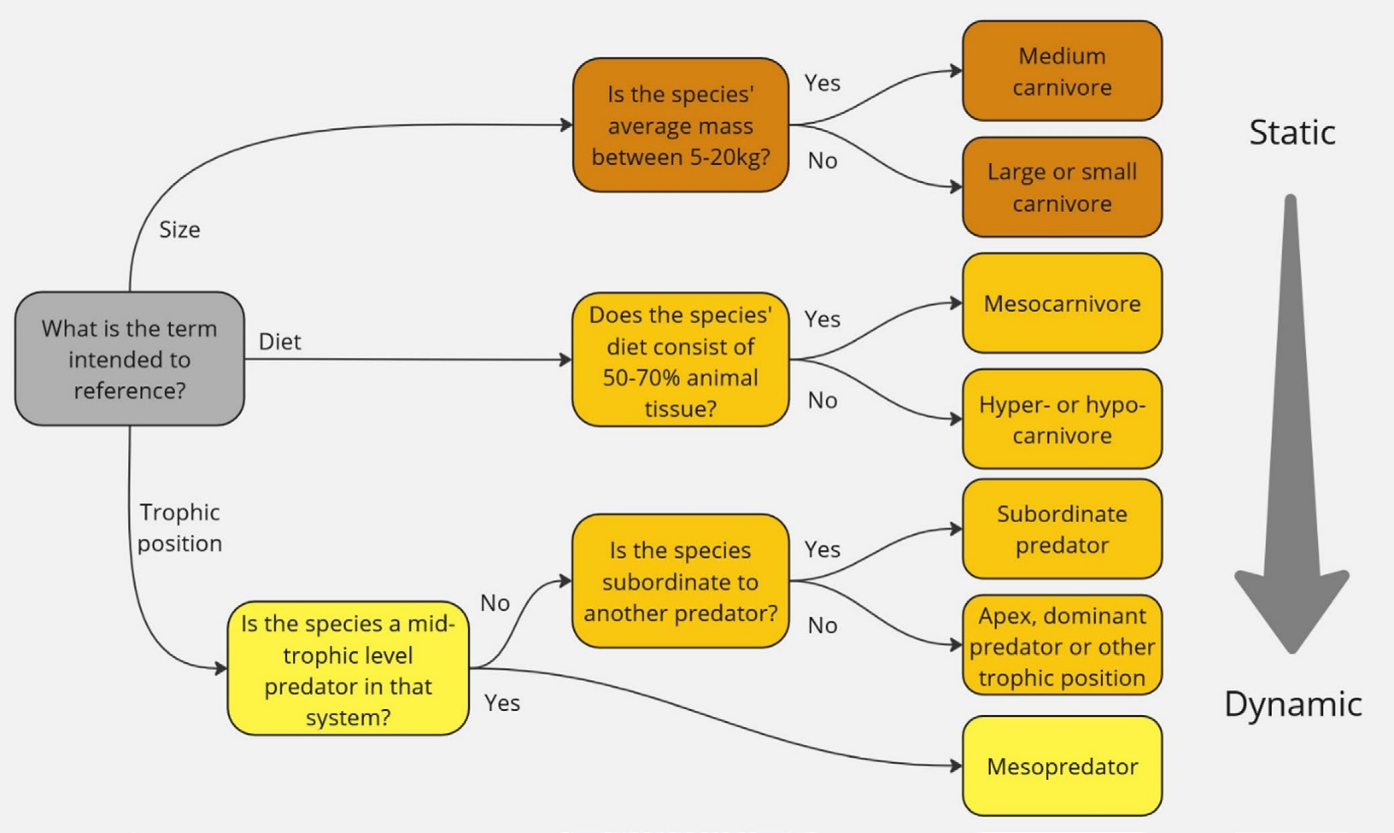
Decision diagram for which term to use to describe a mammalian Carnivore species, depending upon the context of the study, with the darkest orange being the most static, to yellow being the most dynamic. The term ‘mesocarnivore’ should be used strictly for species whose diet consists of 50%–70% animal tissue, independent of their place in the food web, ‘mesopredator’ should be used to refer to a species' mid‐ranking trophic position within a set of predators matching its linguistic meaning. If making pair‐wise comparisons between species such as its relation to an apex predator, then ‘subordinate predator’ is appropriate. If species are being grouped based on size, then it is clearest to use size‐ related terminology.

There has been drift from the original definitions of mesopredator and mesocarnivore, though we believe the original distinctions remain important. Instead of adapting definitions to fit popular usage, we call for precise, context‐appropriate usage. Whilst size‐based descriptors may not translate well across taxa, trophic and dietary terms offer broader relevance. Clear definitions and consistent usage will enhance communication and collaboration among researchers working across taxonomic groups. Restoring the term mesocarnivore to its original dietary context, while reserving mesopredator for use in describing intermediate trophic positioning between two other distinct levels of predators, will help clarify ecological discussions and improve terminological consistency (Figure 2). However, we anticipate that these generalised definitions may not fully accommodate the complexity of many natural systems. In some trophic contexts, if referencing pairwise interactions, subordinate versus dominant predator may be more relevant terms less open to misinterpretation. However, even this can sometimes be ambiguous, so it is important always to clarify which form of dominance (e.g., competitive or physical) is being referenced. Accurate and consistent terminology is paramount in science, and we should strive for consensus on these definitions, ensuring clear communication and accurate representation of each species' role within specific ecosystems.

## Author Contributions

**Emily K. Madsen:** conceptualization (equal), writing – original draft (equal), writing – review and editing (equal). **Lucy Eckersley:** conceptualization (equal), writing – review and editing (equal). **Jennifer F. Linden:** writing – review and editing (equal). **Sandra Lai:** writing – review and editing (equal). **Darragh Hare:** writing – review and editing (equal). **David W. Macdonald:** writing – review and editing (equal). **David Kimaili:** writing – review and editing (equal). **Salum Kulunge:** writing – review and editing (equal). **Yolanda Mutinhuma:** writing – review and editing (equal). **Lisanne Petracca:** writing – review and editing (equal). **Betty J. Rono:** writing – review and editing (equal). **Lovemore Sibanda:** writing – review and editing (equal). **Claudio Sillero‐Zubiri:** writing – review and editing (equal). **Jessica Tacey:** writing – review and editing (equal). **Femke Broekhuis:** conceptualization (equal), writing – review and editing (equal).

## Funding

The authors have nothing to report.

## Conflicts of Interest

The authors declare no conflicts of interest.

## Data Availability

There are no data used in this paper.
